# The Mediation Effect of Phobic Anxiety on the Treatment Outcome of Activity and Participation across Age: Comparison between Online and Face-to-Face Rehabilitation Aftercare of an RCT

**DOI:** 10.3390/ijerph182010919

**Published:** 2021-10-17

**Authors:** Lingling Gao, Alina Dahmen, Franziska Maria Keller, Petra Becker, Sonia Lippke

**Affiliations:** 1 Department of Psychology & Methods, Jacobs University Bremen, 28759 Bremen, Germany; l.gao@jacobs-university.de (L.G.); adahmen@dbkg.de (A.D.); f.keller@jacobs-university.de (F.M.K.); 2 Dr. Becker Klinikgruppe, 50968 Cologne, Germany; pbecker@dbkg.de; 3 Klinikum Wolfsburg, 38440 Wolfsburg, Germany

**Keywords:** Internet- and mobile-based interventions, digital interventions, mental health disorders, phobias, social participation, age, rehabilitation, aftercare

## Abstract

The efficacy of internet and mobile-based interventions (IMIs) has been demonstrated with different mental health disorders, but little is known about the mediating effect of phobic anxiety on activity and participation and the differential effect of age. The current study tested a moderated mediation model with short-term change in phobic anxiety mediating between treatment (IMI vs. face-to-face, F2F) and long-term change in activity and participation, and age of patients moderating this mediation. Participants (*N* = 142) were recruited from psychosomatic rehabilitation clinics and randomized into the IMI psychosomatic aftercare or F2F psychosomatic aftercare. Moderated mediation analyses were conducted using R software. Results showed that the long-term treatment effects of activity and participation (β_c_ = −0.18, *p =* 0.034; β_c’_ = −0.13, *p* = 0.145) were improved through the successful decrease of phobic anxiety (β_a_ = −0.18, *p* = 0.047; β_b_ = 0.37, *p* = 0.010). Older patients benefited equally from both IMI and F2F interventions regarding short-term treatment change in phobic anxiety, while younger participants benefited more from IMI (β_Age*Treatment_ = 0.20, *p* = 0.004). IMIs targeting mental disorders can improve activity and participation along with phobic anxiety, especially in younger individuals. The needs of older patients should be considered with the development and improvement of IMIs.

## 1. Introduction

An increased number of patients have mental health disorders, which requires more effective psychosomatic rehabilitation services [1]. To alleviate the burden of health care systems, Internet- and mobile-based interventions (IMIs) are highly expected to provide service options for individuals due to multiple advantages, such as the quick adaptability to the user’s needs, the possibility to deliver interventions remotely, or to tailor content to a specific patient group as in a face-to-face (F2F) intervention [2,3]. IMIs are key to serve patients in terms of making up for the shortage of health care professionals in the patients’ vicinity as well as improving patients’ mental disorders and social participation even in the face of mobility constraints. At the same time, research has shown that positive outcomes vary strongly due to individuals’ age, relating to their digital health literacy and ability to make positive use of IMIs [4,5]. By comparing IMIs with more traditional interventions such as F2F group therapy, it has been found that age has a profound effect relating to whether patients benefit more from IMIs or F2F interventions. Understanding which individuals benefit most from IMIs is essential in developing and implementing tailored treatment programs for patients with mental disorders. In previous studies, similar improvements across IMIs and F2F treatments in terms of a decrease in symptom intensity were found with regard to mental disorders such as anxiety disorders [6] and specifically phobic anxiety disorder [7]. According to literature, a phobic anxiety disorder, with respect to the International Classification of Disease-10 (ICD-10) manual, is characterized by symptoms of anxiety related to phobias, which has also been termed as phobic anxiety [8]. Thereby, phobic anxiety represents the symptoms associated with a phobic anxiety disorder. However, there is still a lack of studies to evaluate whether further outcomes, for example, social participation, are mediated by the treatment of symptoms related to a phobic anxiety disorder, especially across age populations [7].

Thus, this study investigates the effects of an IMI in comparison to a F2F intervention with regard to activity and participation as an indicator of social participation, which is a crucial outcome. Even though the primary outcome is to evoke a decrease in symptoms of activity and participation difficulties associated with the diagnosis of a phobic anxiety disorder, the question of this secondary data analysis is whether the effects of the IMI and F2F intervention on activity and participation are mediated by a reduction in symptoms associated with a phobic anxiety disorder and whether this differs with regard to age, as previous research has shown that age has a significant impact on Internet usage [9,10,11]. Analyzing this mediation effect is vital and has been previously suggested [7,12], because the improvement of social participation becomes realistic if symptoms of a phobic anxiety disorder are treated. 

### 1.1. Social Participation

“Activity and participation” is one of the most important indicators that displays recovery from mental disorders, with practical meaning for both individuals and society [13]. Furthermore, “Social participation is a key determinant of successful and healthy aging” [14] (p. 2141). According to the explanation of the International Classification of Functioning, Disability, and Health (ICF), the term activity and participation represents the individuals’ execution of tasks/action and the involvement in life situations [15]. Individuals with mental health disorders often have “limitations in activities or capacities” and “restrictions in participation” [15]. The difficulties in activity and participation, for example, lead to patients with workplace phobia regularly applying for long-term sick leave to avoid the phobic anxiety reaction when thinking of or approaching their workplace [16]. 

In a systematic review of 36 studies, social outcomes were specifically synthesized. However, many studies failed to clearly define the social outcomes being addressed [17]. Thus, a validated measure such as activity and participation [18] would be required. The authors of the systematic review state that their “findings suggest that there is a need for more studies that both utilize new and emerging technologies and evaluate their effectiveness in extended field studies and broader user evaluations” [17] (p. 191). Accordingly, this gap is to be closed by the current study, especially because social participation was identified as an area of key importance to be considered when working in Health 2.0, which encourages making use of technologies to promote collaboration between patients and medical professionals [19].

Moreover, in the treatment of mental disorders, the interventions that aim to improve activity and participation need to consider multiple factors, which include social participation, psychological factors like anxiety and specifically phobic anxiety, as well as the interrelations with age [20,21]. Previous studies that considered multiple factors have made efforts on how to promote activity and participation regarding certain mental illnesses or disorders, such as anxiety and depression [22]. However, there is a lack of evidence that explores the mechanisms of whether phobic anxiety mediates the treatment effect of activity and participation, although meta-analyses aim to research such processes [12]. 

### 1.2. Phobic Anxiety 

In fact, phobic anxiety disorder is one of the most common mental disorders, and is usually accompanied by difficulties in activity and participation [23]. There are hundreds of phobias to be found and they can be classified into three subtypes: agoraphobia, social phobia, and simple/specific phobia [24,25]. Individuals with a phobic anxiety disorder, such as agoraphobia, social phobia, or claustrophobia, will reduce normal activity and participation due to the fears of being in open spaces (i.e., with agoraphobia) or going out of the house alone [26]. Moreover, previous studies have found that the comorbidity rates of phobic anxiety disorders and other mental disorders are high [27]. Therefore, the treatment of symptoms related to a phobic anxiety disorder is also of importance for the prevention of other mental disorders [27]. Previous studies have proven that cognitive-behavioral therapy (CBT) is a robust and effective treatment for mental disorders, in terms of decreasing maladaptive behaviors by increasing adaptive ones. Practice is needed for adaptive behaviors in order to result in new learning [28]. Hence, CBT therapy often requires several weeks or months to produce effects. However, the high costs and long waiting times with regard to therapy placement make it difficult to be delivered to all patients in need. Alternative therapy methods, such as one-session treatment (OST) and acceptance and commitment therapy (ACT), have also been shown to significantly support mental health and lead to a decrease in symptomatology compared to a waiting control group or receiving no treatment [29,30]. Hence, these other treatment options provide alternatives for CBT with regard to certain phobic anxiety therapies [29,30]. Besides the common F2F therapy, IMIs are also highly promising to provide a digitalized treatment option for symptoms related to a phobic anxiety disorder, as IMIs are beneficial for the treatment process in terms of reducing costs for health insurance companies as well as providing long-term and location-independent help for many patients who are suffering from a phobic anxiety disorder [31].

Previous studies have proven that IMIs have treatment effects with regard to phobic anxiety disorders such as social phobia [32], flying phobia [33], fear of childbirth [34], fear of public speaking, and fear of small animals [35]. While a number of studies have indicated that IMIs have low adherence rates among patients who display a low motivation on IMIs, some IMIs are suitable for certain patients. For example, avoidant agoraphobic patients benefit from IMIs as they might feel less resistance when seeking help online compared to visiting a therapist [36,37]. The treatment effects of IMIs have been proven for different patients’ characteristics, including mental health status, belief, and attitude to technology, personal traits, and social demographics such as gender and age [38]. 

### 1.3. Age Differences

Although mental disorders are common in the elderly population, they are less likely to receive professional mental health care compared to the younger population [39]. This occurs especially with IMIs since older populations meet more technological barriers due to a lack of skills or not being engaged with mobile devices. Previous studies found that people aged ≤50 years engaged more with IMIs than the older population [9,10,11]. It was also found that a depression diagnosis was negatively related to eHealth literacy scores, which were lower among an older population aged and over 60 [40]. However, in a systematic review, age was not found to be related to treatment effects [9]. A previous systematic review also pointed out that many studies did not investigate age differential effects, although age was considered an important aspect compared to other factors, such as region, race, and personality traits, in the use of IMIs [41,42]. 

**Figure 1:**
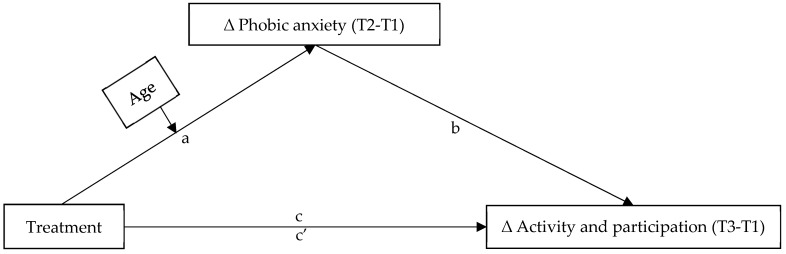
Conceptual model predicting mechanisms of treatment effectiveness in activity and participation mediated by phobic anxiety and moderated by age.

In this study, the role of age was also investigated. We used a randomized controlled trial to examine if IMIs can significantly improve mental health (treatment study) in comparison to F2F therapy across age groups, with secondary data analysis, to gain insights into the mechanisms of IMIs in the treatment of mental disorders and to improve social participation. The research conceptual model predicting mechanisms of treatment effectiveness in activity and participation mediated by phobic anxiety and moderated by age is outlined in Figure 1.

### 1.4. Research Questions

We also aimed to facilitate decisions on further implementation and sustainability of IMIs in routine care settings with the main focus on mechanisms and moderators, i.e., subgroup analyses. This is novel as the primary paper from this data set investigated only the effects in general and in different subgroups, but not the mediating mechanisms. This will increase the understanding of digital evidence-based interventions in the future as well as provide directions for future research. Accordingly, the following hypotheses are examined:

(1) The short-term change in phobic anxiety mediates the relationship between treatment (IMI vs. F2F) and long-term change in activity and participation.

(2) Age of patients moderates the treatment effect of the short-term change in phobic anxiety disorders. 

## 2. Materials and Methods

### 2.1. Procedure

This experimental study used a randomized controlled trial (RCT) design to compare the effectiveness of an online (IMI) and a face-to-face (F2F) rehabilitation aftercare. Rehabilitants who declared their participation in written consent were randomly assigned in a parallel design (1:1) by blinded administrative staff to the intervention (IMI) or the active control group (F2F). Block randomization using permuted variable-length blocks was performed using Randlist software. Double-blinding was not implemented as it was clear to the patient in which group s/he was. 

Outcomes were assessed by online questionnaires at the end of rehabilitation (baseline of aftercare, T1), 12 months after the end of rehabilitation (after completion of aftercare, T2), and 18 months after the end of rehabilitation. At the measurement timepoints of T2 and T3, study participants were invited to complete the questionnaires by email and were reminded 2 and 4 weeks later. The digital platform soscisurvey.de was used for data collection. Data protection and security guidelines of the platform as well as a detailed data protection concept are available.

All processes were tested in a feasibility study before the start of this RCT study. It included ten qualitative interviews with participants of the first groups of the web-based and the F2F follow-up on the usability of the technical application as well as on the therapeutic procedure. No changes were required. The study protocol was published on ResearchGate. The Ethics Committee of the North Rhine Medical Association granted approval for the study design (number of the decision 2015351, and date of approval 04.12.2015). This study was run in a rehabilitation treatment program and aftercare setting. The approval was given by the pension funds (Bund, Braunschweig-Hannover and Rheinland) and corresponding patient councils. The clinical trial registration was retrospectively performed with ClinicalTrials.gov with the name of “Effectiveness and Equivalence of an Internet-based Virtual Classroom Intervention for Psychosomatic Aftercare”.

### 2.2. Participants

Participants were recruited from three psychosomatic rehabilitation clinics from Dr. Becker Klinikgruppe, Germany. During rehabilitation stays at the clinics, patients were asked whether they wanted to take advantage of an aftercare therapy offer. In the case that participants expressed a desire to take part in the study, they were informed in writing about the study conditions. Recruitment took place at the clinics Dr. Becker Klinik Möhnesee March 2017 to May 2018, Dr. Becker Klinik Juliana March 2017 to April 2018, and Dr. Becker Burgklinik March 2017 to September 2020. 

Inclusion criteria for further study participation were an indication for psychosomatic aftercare (indication according to the framework concept of rehabilitation aftercare [43]) and access to a commercially available computer, tablet, or smartphone with broadband Internet connection (DSL or LTE). The exclusion criteria for participation in the study were also according to the framework concept of rehabilitation aftercare [43], these included the ability to perform <3 h/day in the general labor market, receiving or applying for an old-age pension of at least two-thirds of full-time, and receiving a benefit that is regularly paid until the start of an old-age pension (disability pension). Furthermore, patients with acute addictive disorders and psychosis were excluded. 

There were 167 participants included in this study who met the inclusion criteria (randomized to the IMI, *n* = 79 or the F2F, *n* = 88). Among them, 5 participants of the IMI group and 20 participants of the F2F group were found to have violated the study protocol during the follow-up. Excluding those non-adherent participants, 142 participants were analyzed in this study. All participants had been diagnosed at the clinics according to the ICD-10 diagnostic criteria (F20.0-F61 and G43.1). The most frequent diagnoses were F33.1 Recurrent depressive disorder (*n* = 46), F32.1 Depressive episode (*n* = 25), and F43.2 Adjustment disorders (*n* = 21). The mean age of participants was 50.9 years old (SD = 9.28; range 27–67). Among participants, 62% were women (*n* = 88); 54.9% were unmarried (*n* = 78); 76.1% were employed (*n* = 108); and 52.8% had an education level under high school (*n* = 75).

### 2.3. Aftercare Interventions

The aftercare intervention among the F2F participants was based on the concept of the Curriculum Hannover [44,45], which was mainly conducted through group interventions. In addition to the intake and final interview (each a 50-min individual interview), 25 weekly group sessions with a duration of 90 min each and with a group size of 8–10 participants took place. Contents included, for example, how to cope with conflicts at home or the workplace, how to relax, and how to get through daily life. Learning content was delivered by therapists using hard copies and physical whiteboards. 

The IMI aftercare was carried out according to the same concept and timeline [44], but had technical features due to the digital nature: The participants were instructed in advance in the use of the video platform by means of an e-learning video; this also included the rules for communication in the “virtual classroom” as well as for instructions on how to regularly check the digital line. The psychotherapists were prepared for the specifics of the new medium through standardized “train-the-trainer” courses in order to be able to establish a therapeutic relationship in a targeted manner. Learning content was delivered by therapists using PowerPoint presentations or an online whiteboard, accompanied by the distribution of digital handouts or homework. 

There were 12 therapists who participated in the study. All of them were licensed psychotherapists, who worked in rehabilitation clinics or were established as psychotherapists in the outpatient sector.

### 2.4. Measurements

Phobic anxiety was measured by the five-item subscale of the Hamburg Modules for the Assessment of Psychosocial Health in Clinical Practice (HEALTH-49) on a 5-point Likert scale [18]. The HEALTH-49 covers six independent modules from A to F. The measurement of phobic anxiety used a subscale of module A (psychological and somatoform complaints). The example statement was “I fear going out of the house alone” and was scored from 1 (“not at all”) to 5 (“very much”); a lower score of phobic anxiety therefore indicating a better mental health status. The Cronbach’s Alpha in this study was 0.89.

Activity and participation was measured on a 5-point Likert scale by means of 6 items from a subscale of HEALTH-49 (module E) [18]. The example statement was “I couldn’t work as carefully as usual” and was scored from 1 (“never”) to 5 (“always”); a lower score of activity and participation therefore indicating better mental health regarding activity and participation in this study. The Cronbach’s Alpha in this study was 0.89.

Participants also answered socio-demographic questions, such as age (using an open-ended question and categorizing it evenly as a three-level variable), gender, marital status, employment status, and education level (dichotomous variables). 

### 2.5. Dropouts

All 142 participants completed the questionnaire at T1. Among IMI participants (*n* = 74), 30 participants completed the questionnaire at T2 (dropout rate from T1 to T2 was 59.5%), and 26 participants completed the questionnaire at T3 (dropout rate from T1 to T3 was 64.9%). Among F2F participants (*n* = 68), 26 participants completed the questionnaire at T2 (dropout rate from T1 to T2 was 61.8%), and 20 participants completed the questionnaire at T3 (dropout rate from T1 to T3 was 70.6%). There were no significant differences between the participants who completed T2 and those who dropped out at T2 regarding gender (*χ*^2^_142_ = 1.36, *p* = 0.244), age (*F*_1,141_ = 1.03, *p* = 0.311), baseline phobic anxiety (*F*_1,141_ < 0.001, *p* = 0.984), and baseline activity and participation (*F*_1,141_ = 0.12, *p* = 0.726). Furthermore, there were no significant differences between the participants who completed all waves (T1, T2, T3) and those who dropped out at T2 or T3 regarding gender (*χ*^2^_142_ = 0.85, *p* = 0.357), age (*F*_1,141_ = 0.31, *p* = 0.579), baseline phobic anxiety (*F*_1,141_ = 0.03, *p* = 0.853), and baseline activity and participation (*F*_1,141_ = 0.08, *p* = 0.780).

### 2.6. Statistical Analysis

The statistical analyses were performed using R software (Version 4.0.4, Foundation for Statistical Computing, Vienna, Austria). The missing variables were estimated through multiple imputations using the MICE package, which created multiple imputations based on the Fully Conditional Specification method, where each incomplete variable is imputed by a separate model [46]. Previous studies have shown that multiple imputation estimates have the lowest bias compared to other options (e.g., paired exclusion, and regression imputation) if the dropout rate is high in each case [47]. In particular, the MIDAStouch procedure was applied in MICE to relatively lower the bias caused by small sample sizes [48]. The lavaan package in R was used to conduct analyses of a moderated mediation model [49]. Significance was considered statistically accepted at *p* ≤ 0.05.

## 3. Results

### 3.1. Preliminary Analyses 

Overall, 142 participants were included in the study with ages ranging from 27 to 67 years (Table 1). To investigate the treatment effects of phobic anxiety among different age populations, the participants were evenly divided into three age groups: G1 (27–49 years old, *n* = 50), G2 (50–56 years old, *n* = 45), and G3 (57–67 years old, *n* = 47).

The IMI group reported a short-term outcome regarding phobic anxiety (T2) of M = 1.66 (SD = 0.62) and a long-term treatment outcome regarding activity and participation (T3) of M = 2.67 (SD = 0.73). The F2F group reported a short-term outcome regarding phobic anxiety (T2) of M = 1.68 (SD = 0.75) and long-term treatment outcome regarding activity and participation (T3) of M = 2.83 (SD = 0.62). 

### 3.2. Mediation Effects

**Figure 2:**
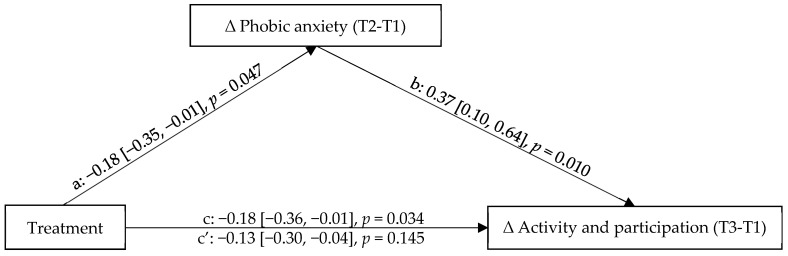
Mediated model with standardized regression coefficients predicting mediation effects of short-term change in phobic anxiety between treatment and long-term change in activity and participation. T1, baseline time point measurement; T2, 12-months follow-up time point measurement; T3, 18-months follow-up time point measurement.

It was investigated whether the short-term change in phobic anxiety (T2-T1, mediator) mediated the relationship between treatment (independent variable) and long-term change in activity and participation (T3-T1, dependent variable) when controlling for gender, marital status, employment status, and education level (control variables). There was no further information on diagnoses included in the model. The mediated model with standardized regression coefficients is depicted in Figure 2. It showed a good model fit, with a ratio of mean chi-square to degrees of freedom (CMIN/DF) = 1.25, Tucker-Lewis index (TLI) = 0.96, and root mean square error of approximation (RMSEA) = 0.04. It revealed that the association between treatment and long-term change of activity and participation (see Figure 2, path c) was significant when controlling for the covariates, indicating that the IMI improved activity and participation more than the F2F intervention. The association between treatment and the short-term change in phobic anxiety (Figure 2, path a) was significant when controlling for the covariates, too. This indicated that the IMI reduced phobic anxiety more than the F2F intervention. 

The association between the treatment and the long-term change in activity and participation became insignificant when including the mediator short-term change in phobic anxiety (T2-T1) (Figure 2, path c′). Thus, it was found that the short-term change in phobic anxiety (T2-T1) fully mediated the relationship between treatment and the long-term change in activity and participation (T3-T1). While the IMI rehabilitation aftercare was associated with a greater decrease in phobic anxiety and activity and participation difficulties compared to F2F rehabilitation aftercare in general, the mediation was as expected: If patients benefitted from the intervention in terms of a reduction in phobic anxiety, then they were also more enabled to adopt activity and participation but the advantage of the IMI treatment on activity and participation disappeared. The patients who were not able to make use of the treatments (indicated by a decrease in phobic anxiety) were also not achieving activity and participation. 

### 3.3. Moderated Mediation Effects

To further test whether the mediation of the short-term change in phobic anxiety (mediator) between treatment (independent variable) and long-term change in activity and participation (dependent variable) differed across age groups (moderator), moderated mediation effects were tested controlling for gender, marital status, employment status, and education level (control variables). There was no further information on diagnoses included in the model. The conceptual model is depicted in Figure 1.

**Figure 3:**
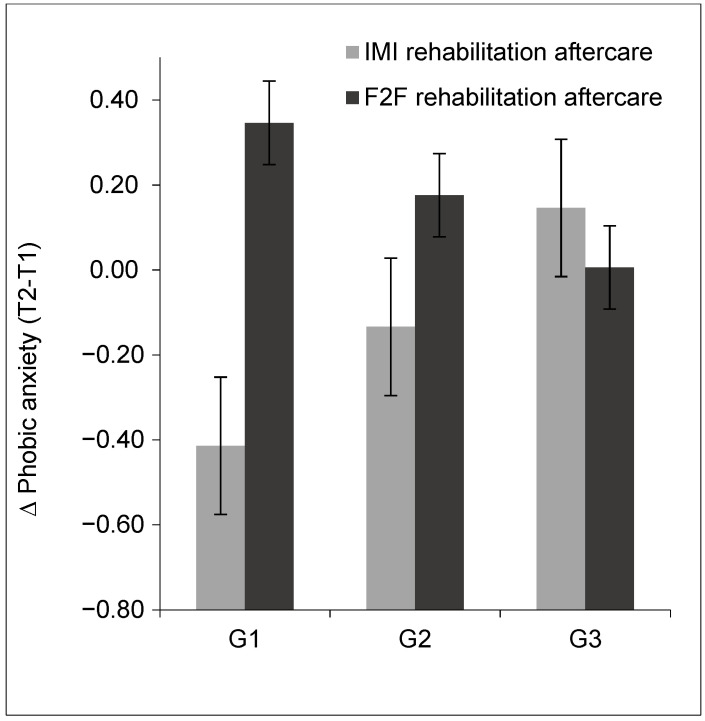
Visualization of the results testing age as a moderator of the short-term treatment change in phobic anxiety in the IMI and the F2F rehabilitation aftercare. G1, 27–49 years old; G2, 50–56 years old; G3, 57–67 years old. T1, baseline time point measurement; T2, 12 months follow-up time point measurement.

The overall moderated mediation model provided a good model fit to the data, with a ratio of mean chi-square to degrees of freedom (CMIN/DF) = 1.24, Tucker-Lewis index (TLI) = 0.90, and root mean square error of approximation (RMSEA) = 0.04. The model was supported with the index of moderated mediation β = 0.11 (95% CI (0.01, 0.21), *p* = 0.033). The results indicated a significant moderating effect of age: the term Age*Treatment had a significant interaction effect with β = 0.20 (95% CI (0.01, 0.38), *p* = 0.004), which indicated that age moderated the relationship between treatment and short-term change in phobic anxiety. The interaction effect was >0 while the treatment effect of short-term phobic anxiety was <0 (Figure 2). The mediation effect of phobic anxiety tended to weaken as age increased. That is to say, the younger participants showed a stronger relationship between treatment and short-term phobic anxiety change and, with that, on activity and participation. Figure 3 displays the results from testing age as a moderator of the short-term treatment change in phobic anxiety between the IMI and F2F rehabilitation aftercare. This short-term treatment change in the IMI rehabilitation aftercare was significantly better in G1 compared to F2F rehabilitation aftercare (*F*_1,46_ = 8.61, *p* = 0.003), which indicates G1 participants benefitted especially from the online treatment regarding short-term treatment change in phobic anxiety. Among G2 and G3 participants, the change in phobic anxiety in IMI rehabilitation aftercare and F2F rehabilitation aftercare did not show significant differences (G2: *F*_1,41_ = 0.30, *p* = 0.588; G3: *F*_1,44_ = 0.16, *p* = 0.689), indicating that the two older groups benefitted equally from both interventions. 

Additionally, the indirect effect within various age groups (setting G1 = 1, G2 = 2, G3 = 3) further revealed the moderated mediation: for participants in G1, the conditional indirect effect was 0.01 (95% CI (−0.14, 0.16)); for participants in G2, the conditional indirect effect was 0.12 (95% CI (−0.11, 0.35)). This means older age weakened the indirect effect from treatment to long-term change in activity and participation via the short-term phobic anxiety change compared to young participants. For participants in G3, the conditional indirect effect was continued to be larger to 0.23 (95% CI (−0.09, 0.55)), which means that the indirect effect from treatment to long-term change in activity and participation via the short-term phobic anxiety change was further diminished. These results indicated that the indirect effect of treatment on the long-term change in activity and participation through the short-term change in phobic anxiety was dependent on the age of patients, such that older patients had a decrease in the indirect effect’s magnitude, i.e., they seemed not to benefit more from the IMI intervention, particularly in increasing activity and participation by decreasing their phobic anxiety, whereas this was not the case for the younger patients.

## 4. Discussion

This study with 142 psychosomatic patients conducted a moderated mediation model to investigate the mechanisms underlying the relationship between treatment and long-term change in activity and participation, with the short-term change in phobic anxiety as a mediator and age as a moderator. Compared to the F2F, the IMI had a greater improvement in activity and participation, which indicated a better treatment outcome. Moreover, the IMI had a better treatment outcome for mental health regarding phobic anxiety with a greater decrease in symptoms compared to F2F. The short-term change in phobic anxiety mediated the relationship between treatment (IMI and F2F) and the long-term change in activity and participation. Furthermore, this mediation differs across age groups, as older patients showed decreased indirect effect magnitude in this process compared to younger patients. 

The mechanisms explored in this study, with short-term treatment change in phobic anxiety mediating the treatment effect on activity and participation, are relatively new. This finding is in line with and adds to previous studies, which reported that phobic anxiety interfered with individuals’ lives and activities [26]. It provides the insight that when identifying and providing treatment to individuals with activity and participation difficulties, phobic anxiety should also be considered as one of the possible barriers to becoming active. While the IMI was able to reduce phobic anxiety and to improve activity and participation in younger patients, IMI appeared to not be more helpful for older patients in comparison to F2F.

Furthermore, phobic anxiety was common among the elderly population and should receive the attention of health professionals [50]. The findings of this study have shown that the older participants (age ranged 50 to 67 years old in this study) benefitted equally from both IMIs and F2F interventions regarding short-term treatment change in phobic anxiety, while younger participants (age ranged 25 to 49 years old in this study) benefit more from IMIs. One possible reason for this finding might be that younger participants have higher self-efficacy, digital literacy, and abilities using online health services compared to older participants [51]. 

Another possible reason for the advantage of IMIs in younger patients might be that many young adults face time conflicts between household activities and their personal activities, such as taking care of small children. For young populations, IMIs provide more flexible time arrangements and less time spent on the way to F2F treatment. Adults around and over 50 years old have relatively more time for personal activities like taking part in therapy (as children might need less time to be accompanied). 

These results are consistent with previous findings that IMIs appear to be non-inferior to F2F treatments for mental disorders; some of which even revealing an advantage in saving time and costs [51,52]. IMIs are becoming increasingly expected to assist face-to-face psychosomatic rehabilitation in terms of making up for the shortage of health care professionals in the area the patients reside in and overcoming the challenges of a pandemic such as COVID-19 [53]. 

When taking this advantage into account, it should be noted that IMI treatments appear more suitable so far for certain patients who benefit from remote treatment, i.e., young participants in this study. This is also supported by previous studies examining age differences in the IMIs [51,54]. Besides age, other variables, such as e-health literacy, might affect the positive outcomes from IMIs; accordingly, such aspects should also be considered in the future [40,55]. Future studies should address older patients specifically in order to meet their needs better. This is especially important because some patients might also face mobility limitations and difficulties in commuting to their psychotherapy or F2F group sessions. 

While this study illuminated the mechanism of the treatment regarding the mediation effect of age, further work needs to be done to better understand how the intervention might work. There is some previous work theorizing that IMIs may work by means of “…Offering ongoing or one-time social and emotional support. …Providing information and psychoeducation to improve informed decision-making. …Improving evidence gathering and documentation. …Improving mental health outcomes (depressive disorders, generalized anxiety disorder…).” [12] (p. 6). Importantly, the authors also conclude, “With the proliferation of mobile devices and the ubiquity of Internet coverage (even in some low-income settings), … digital interventions are considered a cost-saving, provider-mediating, and scalable bargain. Particularly, where they augment conventional modalities by targeting a spectrum of health and psychosocial outcomes related to … victimization, including suicidality, depression, anxiety disorders, PTSD, social withdrawal, low self-esteem and self-efficacy, economic self-sufficiency, and other sequelae” [12] (p. 6). Accordingly, the intervention applied in the current study appears to be such a digital intervention with several potentials. To make better use of IMIs, as previous digital intervention studies have also suggested, meeting the requirements of patients is of high importance for the acceptance and treatment effects of IMIs [12,56]. Individualized digital services are also needed to address the older patient groups more adequately as the needs of older patients are more difficult to imagine but should be actively involved. What exactly older patients need with an IMI remains an open question for future research.

The results of this study should be interpreted with caution due to several limitations. Firstly, the dropout rate was high, and multiple imputations were used to reduce bias. In the future, to lower the dropout rate specific strategies should be adopted, such as providing individually tailored interventions to address the patients’ needs using IMIs. Secondly, this is a secondary analysis with the data being collected for the treatment outcome study, which did not focus on the role of age. Considering sample size and range, age was evenly divided into three groups. The age range was rather small and the individuals over 67 years old were not included at all. Moreover, other socio-demographic variables, such as the number and age of children that patients have, might help to clarify the treatment effect differences and need further attention. Thirdly, other fears were not controlled for as covariant variables, as the main focus of this study was the change in phobic anxiety and its mediation role for activity and participation. The psychosomatic severity was not included. It might affect treatment efficacy and is suggested to be investigated in further research. Finally, this study was conducted in Germany. Due to the differences in mental disorders rehabilitation aftercare, the findings might be different in other countries with other health care systems; thus, replication studies should follow.

## 5. Conclusions

The findings of this study suggest that IMIs can be an effective treatment for phobic anxiety disorders and, with that, improve activity and participation. Older participants benefit equally from both IMIs and F2F interventions regarding short-term treatment change in phobic anxiety, while younger participants benefit especially from IMIs. How to actually accomplish a long-term treatment change with regard to activity and participation through the short-term effects on phobic anxiety in all age groups remains open for future research. This study demonstrates that patients who are using IMIs to improve their mental disorders and overcome symptoms related to a phobic anxiety disorder also benefit in terms of their activity and participation (indicating a full mediation effect), whereas those who do not overcome their phobic anxiety with the provided IMIs presumably require a more tailored IMI to their (age-related) needs and barriers. 

## Figures and Tables

**Table 1:** Descriptive statistics of main study variables at baseline (*N* = 142).

**Variables**	**Face-to-Face Treatment**		**Online Treatment**		**Range**	***t***	***p***	**Cohen’s *d***
	***n***	**M (SD)**	***n***	**M (SD)**				
Age (years)	68	51.10 (9.72)	74	50.70 (8.93)	27–67	0.26	0.798	0.04
G1 (years, *n* = 50)	22	39.10 (6.65)	28	41.32 (5.80)	27–49	−1.27	0.212	0.36
G2 (years, *n* = 45)	21	53.29 (2.24)	24	52.67 (2.06)	50–56	0.97	0.339	0.29
G3 (years, *n* = 47)	25	59.84 (2.19)	22	60.50 (2.60)	57–67	−0.95	0.350	0.28
Phobic anxiety T1	68	1.56 (0.63)	74	1.87 (1.01)	1–5	−2.16	0.032	0.36
Activity and participation T1	68	2.85 (0.89)	74	3.08 (0.96)	1–5	−1.49	0.138	0.25
**Variables**	**Face-to-Face Treatment**		**Online Treatment**			**χ^2^**	***p***	**Cohen’s *h***
	***n***	**%**	***n***	**%**				
Gender						1.18	0.277	0.18
Women	39	57.4%	49	66.2%				
Men	29	42.6%	25	33.8%				
Marital status						3.27	0.071	0.30
Married	36	52.9%	28	37.8%				
Unmarried	32	47.1%	46	62.2%				
Employment status						1.15	0.285	0.18
Employed	49	72.1%	59	79.7%				
Unemployed	19	27.9%	15	20.3%				
Education level						0.133	0.715	0.06
Elementary school	37	54.4%	38	51.4%				
Higher school and above	31	45.6%	36	48.6%				

## Data Availability

The data presented in this study are available on request from the corresponding author.

## References

[1] Petzold M.B., Bendau A., Plag J., Pyrkosch L., Maricic L.M., Betzler F., Rogoll J., Große J., Ströhle A. (2020). Risk, resilience, psychological distress, and anxiety at the beginning of the COVID-19 pandemic in Germany. Brain Behav..

[2] Lippke S., Wienert J., Kuhlmann T., Fink S., Hambrecht R. (2015). Perceived stress, physical activity and motivation: Findings from an internet study. Ann. Sports Med. Res..

[3] Korecka N., Rabenstein R., Pieh C., Stippl P., Barke A., Doering B., Gossmann K., Humer E., Probst T. (2020). Psychotherapy by telephone or internet in Austria and Germany which CBT psychotherapists rate it more comparable to face-to-face psychotherapy in personal contact and have more positive actual experiences compared to previous expectations?. Int. J. Environ. Res. Public Health.

[4] Raney L., Bergman D., Torous J., Hasselberg M. (2017). Digitally driven integrated primary care and behavioral health: How technology can expand access to effective treatment. Curr. Psychiatry Rep..

[5] Poppe L., Plaete J., Huys N., Verloigne M., Deveugele M., De Bourdeaudhuij I., Crombez G. (2018). Process evaluation of an eHealth intervention implemented into general practice: General practitioners’ and patients’ views. Int. J. Environ. Res. Public Health.

[6] Hedman E., Andersson G., Ljótsson B., Andersson E., Rück C., Mörtberg E., Lindefors N. (2011). Internet-based cognitive behavior therapy vs. cognitive behavioral group therapy for social anxiety disorder: A randomized controlled non-inferiority trial. PLoS ONE.

[7] Carlbring P., Andersson G., Cuijpers P., Riper H., Hedman-Lagerlöf E. (2018). Internet-based vs. face-to-face cognitive behavior therapy for psychiatric and somatic disorders: An updated systematic review and meta-analysis. Cogn. Behav. Ther..

[8] WHO (1992). The ICD-10 Classification of Mental and Behavioural Disorders: Clinical Descriptions and Diagnostic Guidelines.

[9] Nguyen H.Q., Carrieri-Kohlman V., Rankin S.H., Slaughter R., Stulbarg M.S. (2004). Internet-based patient education and support interventions: A review of evaluation studies and directions for future research. Comput. Biol. Med..

[10] Kannisto K.A., Korhonen J., Adams C.E., Koivunen M.H., Vahlberg T., Välimäki M.A. (2017). Factors associated with dropout during recruitment and follow-up periods of a mhealth-based randomized controlled trial for mobile.net to encourage treatment adherence for people with serious mental health problems. J. Med. Internet Res..

[11] Ströhle A., Große J., Rogoll J., Betzler F., Maricic L.M., Pyrkosch L., Plag J., Bendau A., Petzold M.B., Ho R.C. (2020). Decision letter for “risk, resilience, psychological distress, and anxiety at the beginning of the COVID-19 pandemic in Germany”. J. Med. Internet Res..

[12] Emezue C., Bloom T.L. (2021). Protocol: Technology-based and digital interventions for intimate partner violence: A meta-analysis and systematic review. Campbell Syst. Rev..

[13] Krupa T., Moll S., Fossey E. (2020). Beyond employment: A broader vision linking activity and participation to health, well-being, and recovery. Psychiatr. Serv..

[14] Levasseur M., Richard L., Gauvin L., Raymond É. (2010). Inventory and analysis of definitions of social participation found in the aging literature: Proposed taxonomy of social activities. Soc. Sci. Med..

[15] World Health Organization (2021). International Classification of Functioning, Disability and Health.

[16] Muschalla B., Linden M. (2014). Workplace phobia, workplace problems, and work ability among primary care patients with chronic mental disorders. J. Am. Board Fam. Med..

[17] Baker S., Warburton J., Waycott J., Batchelor F., Hoang T., Dow B., Ozanne E., Vetere F. (2018). Combatting social isolation and increasing social participation of older adults through the use of technology: A systematic review of existing evidence. Australas. J. Ageing.

[18] Rabung S., Harfst T., Kawski S., Koch U., Wittchen H.-U., Schulz H. (2009). Psychometrische überprüfung einer verkürzten version der »hamburger module zur erfassung allgemeiner aspekte psychosozialer gesundheit für die therapeutische praxis« (HEALTH-49). [Psychometric analysis of a short form of the “Hamburg Modules for the Assessment of Psychosocial Health” (HEALTH-49)]. Zeitschrift Psychosomatische Medizin Psychotherapie.

[19] Hesse B., Hansen D., Finholt T., Munson S., Kellogg W., Thomas J.C. (2010). Social participation in health 2.0. Computer.

[20] Dragioti E., Gerdle B., Levin L.-Å., Bernfort L., Dong H.-J. (2021). Association between participation activities, pain severity, and psychological distress in old age: A population-based study of Swedish older adults. Int. J. Environ. Res. Public Health.

[21] Lippke S., Gao L., Keller F.M., Becker P., Dahmen A. (2021). Adherence with online therapy vs. face-to-face therapy and online therapy vs. care as usual: Secondary analysis of two randomized controlled trials. J. Med. Internet Res..

[22] Grammatikopoulos I., Koutentakis C. (2010). Social activity and participation as determinants of anxiety and depression among elderly in primary care. Ann. Gen. Psychiatry.

[23] Wardenaar K., Lim C., Al-Hamzawi A., Alonso J. (2017). The cross-national epidemiology of specific phobia in the world mental health surveys—CORRIGENDUM. Psychol. Med..

[24] Spitzer R.L., Endicott J., Robins E. (1975). Clinical criteria for psychiatric diagnosis and DSM-III. Am. J. Psychiatry.

[25] Regier D.A., Kuhl E.A., Kupfer D.J. (2013). The DSM-5: Classification and criteria changes. World Psychiatry.

[26] Kendler K.S., Aggen S.H., Werner M., Fried E.I. (2020). A topography of 21 phobic fears: Network analysis in an epidemiological sample of adult twins. Psychol. Med..

[27] Lieb R., Miché M., Gloster A.T., Beesdo-Baum K., Meyer A.H., Wittchen H.-U. (2016). Impact of specific phobia on the risk of onset of mental disorders: A 10-year prospective-longitudinal community study of adolescents and young adults. Depress. Anxiety.

[28] Mansell W. (2007). Coping with Fears and Phobias: A CBT Guide to Understanding and Facing Your Anxieties.

[29] Craske M.G., Niles A.N., Burklund L.J., Wolitzky-Taylor K.B., Vilardaga J.C.P., Arch J.J., Saxbe D.E., Lieberman M.D. (2014). Randomized controlled trial of cognitive behavioral therapy and acceptance and commitment therapy for social phobia: Outcomes and moderators. J. Consult. Clin. Psychol..

[30] Wright B.D., Cooper C., Scott A.J., Tindall L., Ali S., Bee P., Biggs K., Breckman T., Iii T.E.D., Gega L. (2018). Clinical and cost-effectiveness of one-session treatment (OST) versus multisession cognitive-behavioural therapy (CBT) for specific phobias in children: Protocol for a non-inferiority randomised controlled trial. BMJ Open.

[31] Hatemi P.K., McDermott R., Eaves L.J., Kendler K.S., Neale M.C. (2013). Fear as a disposition and an emotional state: A genetic and environmental approach to out-group political preferences. Am. J. Political Sci..

[32] Titov N., Andrews G., Choi I., Schwencke G., Mahoney A. (2008). Shyness 3: Randomized controlled trial of guided versus unguided internet-based cbt for social phobia. Aust. N. Z. J. Psychiatry.

[33] Campos D., Mira A., Bretón-López J., Castilla D., Botella C., Baños R.M., Quero S. (2018). The acceptability of an internet-based exposure treatment for flying phobia with and without therapist guidance: Patients’ expectations, satisfaction, treatment preferences, and usability. Neuropsychiatr. Dis. Treat..

[34] Nieminen K., Andersson G., Wijma B., Ryding E.-L., Wijma K. (2016). Treatment of nulliparous women with severe fear of childbirth via the Internet: A feasibility study. J. Psychosom. Obstet. Gynecol..

[35] Rogers M.A., Lemmen K., Kramer R., Mann J., Chopra V. (2017). Internet-delivered health interventions that work: Systematic review of meta-analyses and evaluation of website availability. J. Med. Internet Res..

[36] Kok R.N., Beekman A.T., Cuijpers P., van Straten A. (2017). Adherence to a web-based pre-treatment for phobias in outpatient clinics. Internet Interv..

[37] Andersson G., Carlbring P., Grimlund A. (2008). Predicting treatment outcome in internet versus face to face treatment of panic disorder. Comput. Hum. Behav..

[38] Borghouts J., Eikey E., Mark G., De Leon C., Schueller S.M., Schneider M., Stadnick N., Zheng K., Mukamel D., Sorkin D.H. (2021). Barriers to and facilitators of user engagement with digital mental health interventions: Systematic review. J. Med. Internet Res..

[39] Cooper C., Bebbington P., McManus S., Meltzer H., Stewart R., Farrell M., King M., Jenkins R., Livingston G. (2010). The treatment of common mental disorders across age groups: Results from the 2007 adult psychiatric morbidity survey. J. Affect. Disord..

[40] Choi N.G., DiNitto D.M. (2013). The digital divide among low-income homebound older adults: Internet use patterns, eHealth literacy, and attitudes toward computer/internet use. J. Med. Internet Res..

[41] Guay C., Auger C., Demers L., Ben Mortenson W., Miller W.C., Gélinas-Bronsard D., Ahmed S. (2017). Components and outcomes of internet-based interventions for caregivers of older adults: Systematic review. J. Med. Internet Res..

[42] Kofmehl J.J. (2017). Online Versus in-Person Therapy: Effect of Client Demographics and Personality Characteristics. Ph.D. Thesis.

[43] Deutsche Rentenversicherung (2015). Framework Concept for After-Care for Medical Rehabilitation According to §15 SGB VI (Last Version Dated 1 July 2019). https://www.deutsche-rentenversicherung.de/DRV/DE/Experten/Infos-fuer-Reha-Einrichtungen/Grundlagen-und-Anforderungen/Konzepte-und-Positionspapiere/konzepte_positionspapiere.html.

[44] Kobelt A., Grosch E. (2005). Indikation zur ambulanten Nachsorge (Curriculum Hannover) in der Psychosomatischen Rehabilitation. [Indication for outpatient aftercare (Curriculum Hannover) in psychosomatic rehabilitation]. Psychotherapeut.

[45] Dahmen A., Gao L., Keller F.M., Lehr D., Becker P., Lippke S. (2021). Wirksamkeit webbasierter psychotherapeutische Nachsorge nach psychosomatischer Rehabilitation—Ein Test in zwei Randomized Controlled Trials [Efficacy of web-based psychotherapeutic follow-up after psychosomatic rehabilitation—A test in two Randomized Controlled Trials]. Die Rehabilitation.

[46] Van Buuren S., Groothuis-Oudshoorn K. (2011). Mice: Multivariate imputation by chained equations in R. J. Stat. Softw..

[47] Jakobsen J.C., Gluud C., Wetterslev J., Winkel P. (2017). When and how should multiple imputation be used for handling missing data in randomised clinical trials—A practical guide with flowcharts. BMC Med. Res. Methodol..

[48] Gaffert P., Meinfelder F., Bosch V. Towards an MI-Proper Predictive Mean Matching. https://www.uni-bamberg.de/fileadmin/uni/fakultaeten/sowi_lehrstuehle/statistik/Personen/Dateien_Florian/properPMM.pdf.

[49] Rosseel Y., Oberski D., Byrnes J., Vanbrabant L., Savalei V., Merkle E., Hallquist M., Rhemtulla M., Katsikatsou M., Barendse M. Package ‘lavaan’. https://cran.r-project.org/web/packages/lavaan/index.html.

[50] Sigström R., Östling S., Karlsson B., Waern M., Gustafson D., Skoog I. (2011). A population-based study on phobic fears and DSM-IV specific phobia in 70-year olds. J. Anxiety Disord..

[51] Paige S.R., Miller M.D., Krieger J.L., Stellefson M., Cheong J. (2018). Electronic health literacy across the lifespan: Measurement invariance study. J. Med. Internet Res..

[52] Axelsson E., Andersson E., Ljótsson B., Björkander D., Hedman-Lagerlöf M., Hedman-Lagerlöf E. (2020). Effect of internet vs. face-to-face cognitive behavior therapy for health anxiety: A randomized noninferiority clinical trial. JAMA Psychiatry.

[53] Salawu A., Green A., Crooks M.G., Brixey N., Ross D.H., Sivan M. (2020). A Proposal for multidisciplinary tele-rehabilitation in the assessment and rehabilitation of COVID-19 survivors. Int. J. Environ. Res. Public Health.

[54] Magnezi R., Bergman Y.S., Grosberg D. (2014). Online activity and participation in treatment affects the perceived efficacy of social health networks among patients with chronic illness. J. Med. Internet Res..

[55] Fonseca A., Osma J. (2021). Using information and communication technologies (ICT) for mental health prevention and treatment. Int. J. Environ. Res. Public Health.

[56] Solomou I., Constantinidou F. (2020). Prevalence and predictors of anxiety and depression symptoms during the COVID-19 pandemic and compliance with precautionary measures: Age and sex matter. Int. J. Environ. Res. Public Health.

